# Medicolegal Pitfalls in Management of Shoulder Dislocations: A Case Series

**DOI:** 10.5811/cpcem.53890

**Published:** 2026-09-08

**Authors:** Clara Billings, Abby Bardwell, Aaron Thomas, Rachel Lindor

**Affiliations:** * Des Moines University, College of Osteopathic Medicine, Des Moines, Iowa; † Mayo Clinic, Department of Emergency Medicine, Rochester, Minnesota

## Abstract

**Introduction:**

Shoulder dislocations comprise approximately half of all major joint dislocations evaluated in the emergency department. Despite often being considered a simple injury, diagnosing a dislocation can be difficult, especially when accompanied by a fracture associated with unusual mechanism, or when the physical examination is limited. Missed and delayed diagnoses can result in poor patient outcomes and legal risks for clinicians, necessitating a high level of vigilance.

**Case Series:**

We review three medical malpractice cases centered on the failure to recognize shoulder dislocations. Key issues included missed diagnosis, limited physical examination documentation, and inadequate discharge instructions.

**Conclusion:**

Shoulder dislocations can be challenging to identify, and missed diagnoses give rise to subsequent clinical and legal risks. Emergency physicians must maintain a high index of suspicion for this injury, recognize the limitations of standard imaging, and thoroughly document clinical decision-making during both initial evaluation and patient discharge.

## INTRODUCTION

Shoulder dislocations constitute nearly half of all major joint dislocations evaluated in the emergency department (ED), with anterior displacement representing up to 98% of these shoulder injuries.^1^ The glenohumeral joint is predisposed to instability due to its shallow glenoid fossa, where only about 20% of the humeral head has articular contact with the scapula, as well as a relatively loose joint capsule and ligaments.^1^ Patients with shoulder dislocations often present with severe pain in the shoulder and limited range of motion. Patients with anterior dislocations usually hold their arm in abduction and external rotation, while those with posterior dislocations may internally rotate and adduct their arm. The empty glenoid fossa may be palpated behind the humeral head on anterior dislocations, with the opposite finding in posterior dislocations.

Although plain radiographs are often relied on for diagnosis, they have a relatively low sensitivity for associated fractures and posterior dislocations, necessitating thoughtful physical examination and imaging plans. The standard anterior-posterior shoulder radiograph is often insufficient for identifying humeral displacement, especially posterior dislocations, which are missed on most initial ED evaluations.^2^ The American College of Sports Medicine and the American Medical Society for Sports Medicine recommend, at a minimum, that true anterior-posterior and axillary views be obtained when imaging for shoulder dislocations.^3^ When axillary views are difficult to obtain due to pain or positioning, a scapular Y view or Velpeau view can be useful.^4^ Computed tomography or magnetic resonance imaging are increasingly used in clinical practice when radiographs are inconclusive or when there are suspected associated fractures.^3^

Axillary nerve injury in the setting of shoulder dislocations has been reported in up to 60% of cases.^5^ This is due to the vulnerability of the nerve in its course around the surgical neck of the humerus just inferior to the glenohumeral joint. Most injuries result in deltoid muscle weakness and sensory loss over the lateral shoulder. While most of these heal with time, deficits can be persistent, particularly with delayed diagnosis and treatment.^5^

Here we discuss three medical malpractice cases regarding shoulder dislocations, emphasizing common diagnostic oversights and outlining preventive strategies clinicians can employ to reduce the risk of malpractice litigation. The information reported is from actual verdicts and settlements and, thus, is limited to what was provided by the court reporters.

## CASE SERIES

### Poor Sensitivity of Radiographs

#### Case 1: *Granger*

A male patient presented to the ED complaining of left shoulder pain. He underwent a radiograph and was informed that his shoulder was not fractured. He was placed in a sling and discharged home. The patient continued to experience pain and limited mobility, and approximately three months later he was diagnosed with a shoulder dislocation, ultimately requiring surgical intervention. He filed a malpractice suit against both the emergency physician and the radiologist, alleging negligence for failure to obtain appropriate radiographs, misinterpretation of the radiograph, and inadequate discharge instructions. The case was settled for $543,000.^6^

### Posterior Shoulder Dislocations

#### Case 2: *Magstadt*

A 45-year-old real estate agent presented to the ED endorsing severe right arm and shoulder pain after a fall. The emergency physician suspected a proximal humerus fracture and ordered radiographs, which were read by the radiologist as negative for fracture. The emergency physician diagnosed the patient with a muscle sprain and discharged him. The patient sought a second opinion due to persistent severe pain and was diagnosed with a posterior shoulder dislocation, requiring surgery and resulting in chronic pain and debility. The patient sued both the radiologist and the initial emergency physician, alleging that he should have examined his shoulder to identify the dislocation, performed range of motion tests to recognize the shoulder joint was affected, diagnosed the dislocation based on the radiographs available, and that he should have provided better follow-up instructions for the patient given his severe, persistent pain at discharge. The case ultimately concluded in favor of the physicians, but it required three years and a full jury trial to resolve.^7^

### Concomitant Fracture with Anterior Shoulder Dislocation

#### Case 3: *Stanard*

A 58-year-old woman presented to the ED with right shoulder pain. The radiologist noted a humerus fracture but did not note the associated dislocation that was also present. The patient developed avascular necrosis, chronic pain, and joint instability, and she sued both the emergency physician and the radiologist for their failure to diagnose her dislocation. The emergency physician defended himself by noting that he relied on the report of the radiologist, which noted only the fracture. Six years after the incident and following a jury trial, the case concluded in favor of the physicians.^8^

## DISCUSSION

Diagnosing shoulder dislocations in the ED can be surprisingly challenging. The malpractice cases highlight several common scenarios that cause difficulty for clinicians—namely, poorly controlled pain or concomitant fractures—both of which can complicate the imaging options or exam findings, and the presence of a posterior dislocation, which presents more subtly and requires a higher index of suspicion.

### Exam Findings Associated with Joint Dislocations

There are multiple exam findings and mechanisms of injury that should increase the suspicion of a glenohumeral joint dislocation, indicating the need for additional imaging or urgent referral even when optimal imaging is not readily available. For anterior dislocations, falls on an outstretched hand or blunt trauma to an abducted, externally rotated arm are common mechanisms of injury. Patients may present with a physical deformity including noticeable fullness of the axillary region and/or a prominent acromion.^1^,^3^ Posterior dislocations are classically associated with seizures or electrocutions, although the vast majority are secondary to trauma, specifically when the shoulder is internally rotated and forced into flexion and adduction. A patient’s physical exam may reveal a posterior prominence, flattening of the anterior shoulder, and an inability to externally rotate the arm.^2^

### Appropriate Imaging

The need for plain radiographs with multiple views is imperative during the initial evaluation of all traumatic shoulder injuries. The anterior-posterior radiographic view is useful for evaluating superior-inferior displacement of the humeral head; however, it does not adequately assess anterior-posterior translation. An orthogonal view, such as the axillary view, which requires arm abduction, is essential for accurately identifying shoulder dislocations, particularly in cases of posterior dislocation. Obtaining an axillary view is often difficult due to limited patient cooperation because of significant pain from the dislocation itself, limiting abduction. Consequently, this view is frequently omitted even when explicitly ordered, leading to a potential missed diagnosis.^9^ A modified axial projection, such as the Velpeau view, which does not require abduction, can be useful in these situations.^4^

While anterior shoulder dislocations are less often missed due to their characteristic symptoms, signs, and functional limitations, an accurate diagnosis requires maintaining an appropriate index of suspicion, paired with the proper physical examination and radiographic views.^10^ In Case 1, the emergency physician did not suspect a dislocation and relied on plain radiographs to evaluate the patient for a fracture, with angles potentially limited by pain, ultimately missing an anterior dislocation and resulting in a settlement of $543,000—a reflection of the serious complications that can result from delayed diagnosis.^6^ Missed dislocations can lead to chronic instability, persistent pain, and irreversible loss of function, which in turn may contribute to decreased earning capacity and long-term disability for the patient. As chronic dislocations progress, the likelihood of successful closed reduction diminishes significantly, thereby increasing the need for operative intervention.^10^ This not only elevates the cost of care but also increases patient morbidity and the potential for legal action.

### Posterior Dislocations

Unlike anterior dislocations, posterior dislocations can be easily missed due to their subtle clinical signs, less overt radiology findings, and overlapping symptoms with other shoulder injuries, including rotator cuff tears, ligament sprains, and shoulder contusions.^2^ In Case 2, the physicians did suspect a shoulder dislocation and obtained radiographs to evaluate.^7^ It is unclear what radiographic views were obtained, but the diagnosis of a posterior dislocation was missed. The patient ended up with chronic symptoms, and the physician faced a multi-year lawsuit. It is suggested that posterior dislocations are overlooked up to 80% of the time. This low sensitivity underscores the importance of performing a thorough history and physical exam, which could highlight injury patterns, range of motion, or anatomical nuances suggestive of posterior dislocations, in addition to obtaining appropriate imaging. In patients with the appropriate mechanism of injury and exam findings, clinicians should have a low threshold for computed tomography to further evaluate the shoulder.^11^

### Concomitant Fractures

Failing to recognize dislocations alongside significant fractures highlights another risk of shoulder dislocation management. This is illustrated in Case 3, in which the emergency physician discharged a patient with a proximal humerus fracture but failed to recognize his concomitant dislocation, resulting in delayed diagnosis and treatment. The radiological term “satisfaction of search” refers to the tendency of clinicians to stop thoroughly examining imaging after an initial abnormality is found, potentially causing them to miss other coexisting injuries.^9^ For example, there may be a mistaken belief that if the patient has a proximal humerus fracture, this injury alone appropriately explains the patient’s presentation and symptoms. However, concomitant fracture-dislocations are not uncommon, and finding one should raise suspicions for the other. Clinicians should be cautious not to prematurely conclude the diagnostic process once a single shoulder injury is identified.

## LEGAL LESSONS

Regardless of the cause, failure to identify and reduce a shoulder dislocation in a timely manner can result in significant patient morbidity. Chronic unreduced dislocations are associated with a range of complications including degenerative joint changes, disruption of soft tissue stabilizers, osteoarthritis, and avascular necrosis of the humeral head.^2^ While the best strategy for risk reduction is certainly to never miss a diagnosis, documentation of thoughtful care may be the second-best step. This starts with full documentation of the patient’s history and clinical exam, with special attention to ruling out any neurovascular deficits. In addition, the medical record should reflect that the clinician has considered and evaluated, to the extent they think it reasonable, for the commonly missed injuries such as posterior dislocations and concomitant fractures, when the mechanism calls for it.

Finally, especially in patients who have residual pain, poor function, or other exam limitations at discharge, clear patient education and thorough, written discharge instructions are critical components of risk mitigation. In all cases presented here, plaintiffs cited inadequate post-discharge planning and insufficient follow-up instructions as contributing factors in their malpractice claims.^6^–^8^ Clinicians have a responsibility to ensure patients understand their test results, are aware of signs that should prompt reassessment, and receive clear guidance on symptom management between visits. Spending time to document care and to both discuss and record discharge instructions and follow-up plans can significantly mitigate the risk of future legal claims, even in the face of inevitable clinical uncertainty.

## CONCLUSION

Shoulder dislocations are the most common major joint dislocation seen in the ED. Conducting a thorough physical examination and obtaining appropriate, comprehensive imaging are critical steps in minimizing the risk of diagnostic oversight. Our examination of three malpractice cases related to shoulder dislocations illustrates the diagnostic complexities and management pitfalls that can lead to adverse outcomes and legal action. Enhancing clinical awareness of these challenges can support more effective patient care, improve patient outcomes, and help minimize legal exposure.
